# A Case Report of Endoscopic Retrograde Cholangiopancreatography (ERCP) and Acute Pancreatitis Induced Takotsubo Cardiomyopathy (TCM) in a Patient With Gallstones Induced Acute Pancreatitis and Cholangitis

**DOI:** 10.7759/cureus.24708

**Published:** 2022-05-03

**Authors:** Zahid Khan

**Affiliations:** 1 Acute Medicine, Mid and South Essex NHS Foundation Trust, Southend on Sea, GBR; 2 Cardiology and General Medicine, Barking, Havering and Redbridge University Hospitals NHS Trust, London, GBR; 3 Cardiology, Royal Free Hospital, London, GBR

**Keywords:** st-elevation myocardial infarction (stemi), apical ballooning, transthoracic echocardiogram, gallstone cholecystitis, cardiac chest pain, cardiac troponin, tako-tsubo cardiomyopathy (ttc), common bile duct (cbd), gall bladder diseases and gallstones, endoscopy ercp

## Abstract

We present a case of a 30-year-old-patient, previously fit and well, with abdominal pain radiating to the back for a day. Computerized tomography scan of abdomen and pelvis showed gall bladder calculi and distal common bile duct (CBD) stones. Lab tests showed raised inflammatory markers including high amylase level, so she was treated for gallstones-induced pancreatitis. The patient underwent successful endoscopic retrograde cholangiopancreatography (ERCP) and was discharged. She presented to hospital the next day with severe central chest pain. ST segment elevation was detected on her electrocardiogram in inferolateral leads and ST segment depression in anterior leads. Her echocardiogram showed apical ballooning and blood tests showed elevated troponin T levels. The patient was given aspirin 300 mg and ticagrelor 180 mg stat, and morphine 5 mg intravenously. She also underwent coronary angiogram, which turned out to be normal. The patient was treated for ERCP-induced Takotsubo cardiomyopathy (TCM) and was treated with fluids and antibiotics. She made complete recovery and was discharged home with outpatient follow up.

## Introduction

Takotsubo cardiomyopathy (TCM) is characterized by a transient left ventricular systolic dysfunction (LVSD), electrocardiographic features of acute myocardial infarction (AMI), elevated troponin, and normal coronary arteries on coronary angiogram [1]. It is also known as stress cardiomyopathy, broken heart syndrome, or apical ballooning syndrome. In this condition, the echocardiogram shows diffuse wall motion abnormalities with impaired LVSD in absence of coronary artery disease [2]. TCM was first described by Japanese scientists in 1991, and the name was given to it in view of the distinctive echocardiographic resemblance to traditional takotsubo, or octopus fishing pots [3]. TCM has gained significant attention recently due to impaired LVSD as the event is usually triggered by emotional or physical stress and patients present with typical chest pain with elevated cardiac enzymes [3]. 

The three possible mechanisms for this TCM include catecholamine-induced neurogenic cardiotoxicity, coronary microvascular impairment, and multi-vessel epicardial coronary artery vasospasm [3,4]. Endoscopic retrograde cholangiopancreatography (ERCP)-associated cardiopulmonary complications are reported to be about 16% [5]. The reported cardiac complications incidence secondary to ERCP is lower in large prospective studies (0.07-2.4%) compared to smaller single-center studies. This discrepancy in rates seems to be due to lack of consensus definitions and inconsistent documentation [5]. 

ERCP-related common cardiac problems are mostly minor and include arrhythmias, blood pressure fluctuation, and increased oxygen requirement [5]. Till to date, only seven cases of ERCP-induced TCM have been reported in literature [2]. We present a case of ERCP-induced TCM in a 30-year-old patient with gallstones pancreatitis.

## Case presentation

A 30-year-old-patient five months postpartum, with past medical history of previous gallstones and common bile duct (CBD) calculi, presented with severe abdominal pain radiating to the back associated with nausea and vomiting. On clinical examination, she was found to have generalized abdominal tenderness, was jaundiced, and had dark color urine for the last 48 hours. Lab tests showed raised inflammatory markers as shown in Table 1. 

**Table 1:** Laboratory tests results trend

Investigation	Day 1	Day 2	Day 5	Day 7	Normal value
Hemoglobin	166	115	112	116	110 – 150 g/L
White cell count	22.42	14.34	11.38	8.4	3.5 – 11 x 10^9^/L
Neutrophil count	19.68	9.16	11.52	8.40	1.7 – 7.5 x 10^9^/L
Platelet	463	277	362	388	140 – 400 x 10^9^/L
Sodium	130	135	139	138	135 – 145 mmol/L
Potassium	3.9	4.4	4.5	4.8	3.5 – 5.1 mmol/L
Urea	5.0	5.6	4.2	4.0	2.5 – 7.1 mmol/L
Creatinine	63	36	48	42	49 – 92 umol/L
C-reactive protein	118	178	329	139	0 – 21 umol/L
Amylase	1,524	980	850	200	10 – 100 unit/L
Troponin T	5	2666	2689	3965	< 14 ng/L
Alanine aminotransferase level	177	56	85	60	10 – 35 unit/L
Alkaline phosphatase level	247	120	127	121	0 – 129 unit/L
Bilirubin	118	145	65	45	0 – 21umol/L
Aspartate aminotransferase level	52	159	216	63	10 – 35 unit/L

**Figure 1:**
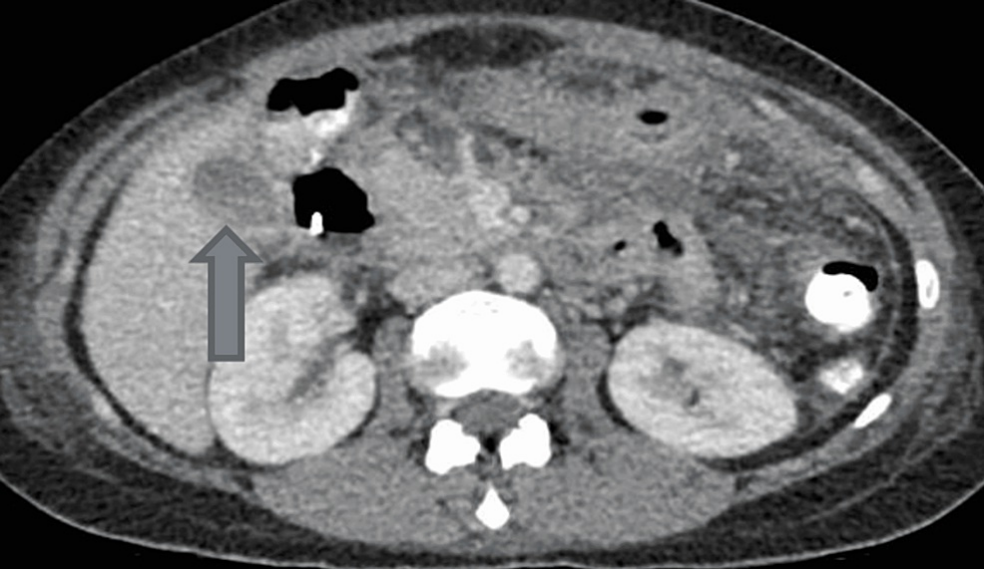
Computerized tomography scan of abdomen and pelvis, showing gallbladder calculus

**Figure 2:**
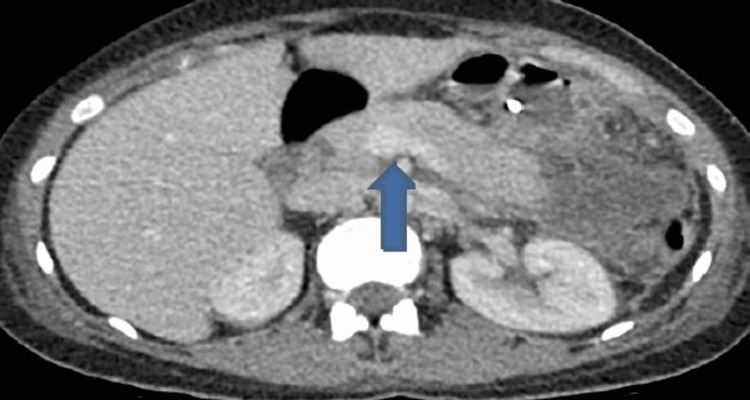
Computerized tomography scan of abdomen and pelvis, showing swollen and oedematous pancreatitis consistent with acute pancreatitis

**Figure 3:**
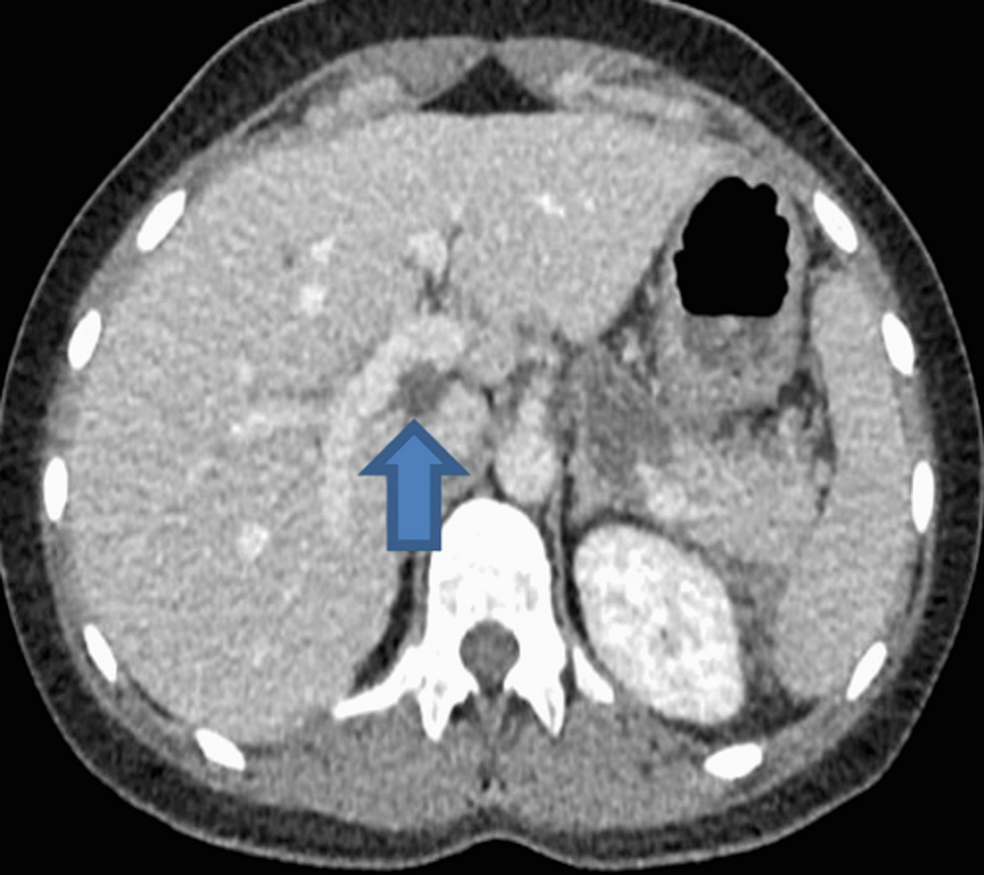
Computerized tomography scan of abdomen and pelvis, showing common bile duct (CBD) stone

Computerized tomography scan of abdomen and pelvis showed features of acute pancreatitis without any evidence of pancreatic necrosis and thin-walled distended gallbladder containing multiple gallstones. The distal CBD was dilated measuring up to 13 mm with positive double duct sign (moderate intra- and extra-hepatic duct dilatation and mild pancreatic duct dilatation) as shown in Figures 1-3. 

**Figure 4:**
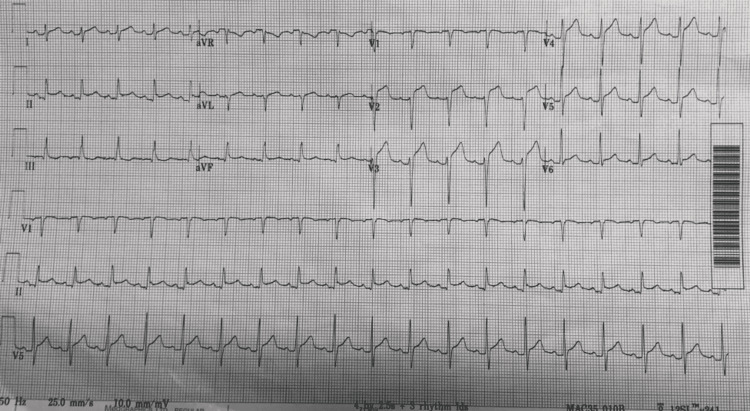
Electrocardiogram showing antero-lateral ST segment elevation

Patient underwent urgent ERCP-guided sphincterotomy to retract the distal CBD calculus. Patient was transferred to the gastroenterology ward following the procedure and was kept nil by mouth for two hours. She developed severe central chest pain the next morning with new ST segment elevation on electrocardiogram (Figure 4) and underwent emergency primary coronary angiography.

Coronary angiogram showed normal coronary arteries and echocardiogram showed apical ballooning of the left ventricle with preserved biventricular function as shown in Videos 1-4. 

**Video 1:** Coronary angiogram shows normal left coronary arteries

**Video 2:** Coronary angiogram shows normal right coronary artery

**Video 3:** Echocardiography—Apical 4 chambers view showing apical ballooning with preserved left ventricular ejection fraction

**Video 4:** Apical ballooning with preserved biventricular function on Apical 4 chambers view

The patient was managed conservatively and was given antibiotics. She was also commenced on dual anti-platelet (DAPT) (aspirin and clopidogrel) initially. However, clopidogrel was stopped later on. Troponin T was elevated despite normal coronary arteries on coronary angiogram, and the echocardiogram showed preserved left ventricular function with apical ballooning consistent with TCM. The inflammatory markers improved over the next few days and troponin T levels showed a downward trend after initial rise. Cardiac monitor showed sinus arrhythmia post-ERCP, and in view of TCM, the patient was commenced on metoprolol 12.5 mg three times a day (TDS) initially, which was then increased to 25 mg TDS due to ongoing tachycardia. Repeat computerized tomography of abdomen and pelvis five days post procedure showed left-sided pleural effusion and oedematous pancreas with overall improvement in the severity of pancreatitis. Cardiac magnetic resonance (CMR) imaging showed extensive sub-epicardial late gadolinium enhancement (LGE) in the lateral wall from base to apex, extending to the inferior and anterior walls and into the mid-distal septum. These findings may be in keeping with an extensive acute myocarditis with preserved biventricular systolic function or TCM. In view of the distribution and extension of the LGE, an overlap with a primary heart muscle disease such as arrhythmogenic left ventricular cardiomyopathy (ALVC) should be considered in the differential diagnosis. The patient stayed in the hospital for a week and was discharged, with outpatient cardiology and gastroenterology follow up. An outpatient surgical referral was sent for this for consideration of laparoscopic cholecystectomy. A repeat echocardiogram prior to discharge showed preserved LVEF.

## Discussion

Historically, TCM cases have been linked to emotional stress. However, in reality, TCM is more commonly associated with physical stress. It is characterized by transient left ventricular wall abnormality resulting in apical ballooning and the main patho-physiological response to stress is increased catecholamine release [6]. TCM was first described by Dote et al. in 1991, who identified five patients to have developed TCM. It was also considered to be more common in Asian population [7]. However, lately, only 2% of patients admitted to hospital with suspected AMI have been found to have TCM [7]. 

TCM is more common in post-menopausal women in their sixties and seventies, and the average age is between 60 and 75 years [7]. Generally, patients with TCM tend to have fewer cardiovascular risk factors such as hyperlipidaemia, hypertension, diabetes mellitus, and positive family history [7,8]. However, one study reported higher incidence of TCM in post-menopausal women with higher cardiovascular risk factors in United States [9]. Studies have shown that the product of peak troponin I levels and LVEF can be used to distinguish between AMI and TCM due to association of TCM with increased level of brain natriuretic peptide levels compared with ST-elevation myocardial infarction (STEMI) [10]. This study also reported that troponin-LVEF product was lower in TCM patients in comparison to STEMI patients [10,11]. 

The cardiac enzymes in TCM patients typically demonstrate mild elevation compared to AMI and return to baseline faster [12]. The Ramaraj et al. study, based on 114 Takotsubo patients, reported that troponin T levels were 6 ng/mL or less and troponin I levels were 15 ng/mL or less in TCM patients [12].

TCM is very rarely associated with acute pancreatitis; the association was first described in 2007 [13]. Only 11 cases of acute pancreatitis associated TCM have been reported since then, and of these 11 patients, nine were female with a median age of 63 years and 82% of cases were aged > 50 years [6]. It can be very challenging to diagnose TCM in patients with acute pancreatitis clinically if the only symptom is epigastric pain, which can can be considered to be due to acute pancreatitis [13]. Patients can only be diagnosed with TCM after having a normal coronary angiogram and echocardiogram showing typical apical ballooning of the left ventricle. Moreover, they may also have significant ECG changes such as ST segment elevation along with elevated troponin [12,13]. The prognosis of TCM associated with acute pancreatitis is excellent and no deaths have been reported so far including in the two cases where patients had cardiac arrests [14]. The LVEF in most patients with TCM returns to normal between 10 days to six weeks. However, Radoslaw et al. reported that LV function may not return to normal based on long-term follow up [13,14]. 

## Conclusions

In conclusion, TCM is common in post-menopausal women and very rarely associated with acute pancreatitis. Patients may develop TCM due to either emotional and/or physical stress. In our case report, this patient developed TCM after undergoing ERCP for cholangitis and also had gallstones-induced acute pancreatitis. Coronary angiogram is normal in patients with TCM, and it is the echocardiogram that usually shows apical ballooning with left ventricular impairment. This patient made a complete recovery and repeat echocardiogram showed normal left ventricular function. 
